# COVID-19 pandemic and civil unrests in Africa: implication of recent #EndSARS protests for increased community transmission in Nigeria

**DOI:** 10.11604/pamj.supp.2020.37.1.26956

**Published:** 2020-12-15

**Authors:** Chinenyenwa Ohia, Mobolaji Modinat Salawu

**Affiliations:** 1 Department of Environmental Health Sciences, Faculty of Public Health, College of Medicine, University of Ibadan, Ibadan, Nigeria,; 2 Department of Epidemiology and Medical Statistics, Faculty of Public Health, College of Medicine, University of Ibadan, Ibadan, Nigeria

**Keywords:** COVID-19 pandemic, #EndSARS protest, community transmission, Africa, youths

## Abstract

The recent Coronavirus disease (COVID-19) caused by the novel strain of coronavirus (SARS-CoV-2 virus) has become a worldwide public health crisis with associated high mortality rates globally. Human to human transmission of the infection emphasizes the importance of adhering to public and social measure to halt its spread. However, the recent #ENDSARS protests led by angry youths in Nigeria resulted to complete flouting of all WHO guidelines meted to curb the spread of the virus. Given that the nation is the most populous African country with over 50 percent of her population been youths, this situation has huge implications for the country and Africa as a whole. The risk of community transmission occasioned by the protest, coupled with travels and active mobility across countries within the continent increases the risk of community transmission with possible increases in new cases over the next few weeks to months. From the foregoing, it is expedient to increase awareness and enforcement of the use of personal protective equipment especially use of nose masks, face shields and frequent hand washing or sanitizing in public places. These individual-targeted measures will go a long way to curtail the imminent community transmission of COVID-19 across Nigeria. It is therefore recommended that Government and non-governmental agencies across the region actively engage to increase collaborative efforts; screening facilities and access to these services across the country, as well as accentuate regular adherence to preventive measures to the general public.

## Perspectives

The Coronavirus Disease (COVID-19) is a respiratory disease which is primarily transmitted between people through droplets and contact routes causing mild respiratory symptoms to severe pneumonia with a fatality rate of about 2% [1,2]. Over 50 million people have been infected during the ongoing pandemic and this has resulted in over 1.2 million deaths globally of which majority are recorded in the region of the Americas [3]. The African region has recorded over 1.9 million cases and over 45,000 deaths with South Africa and Ethiopia reporting highest numbers of new cases, followed by Mozambique, Uganda and Nigeria [3,4]. Currently, Nigeria has recorded over 64, 000 cases of COVID-19 with over 1,100 deaths as at 12^th^ November, 2020 [5]. Several countries including Nigeria have implemented extensive public health preventive measures spanning both pharmaceutical and non-pharmaceutical interventions such as lockdown, closure of borders and social distancing to reduce the surge in imported cases and local transmission of the disease [6]. In spite of this, community transmission is on the rise in Nigeria with new cases been recorded every week. This is suggested to be due to human to human transmission facilitated through respiratory droplets when an infected person coughs or sneezes [1,5].

**Figure 1:**
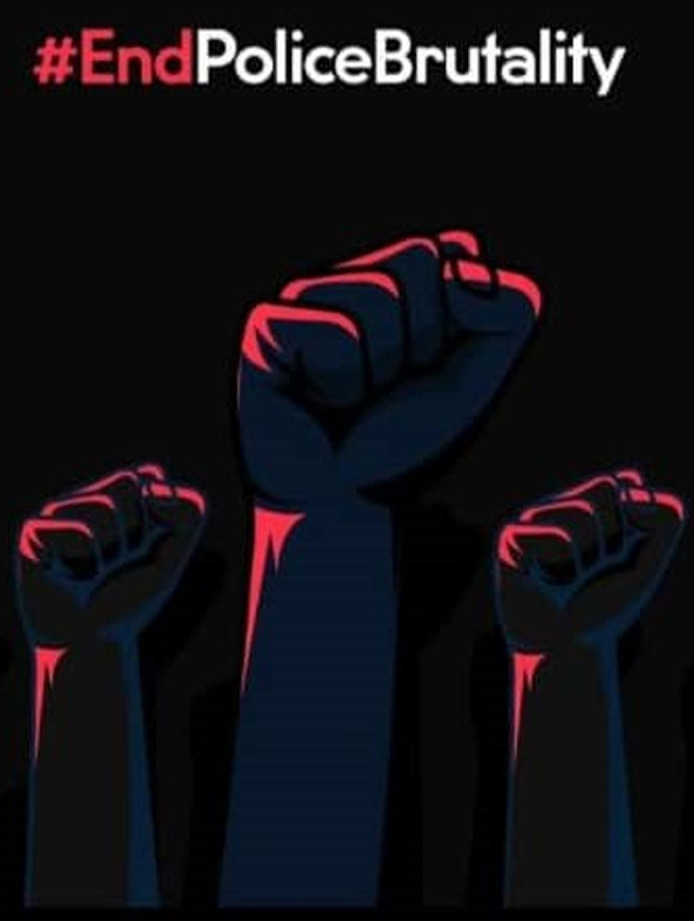
a slogan used during the EndSARS protests across Nigeria

The recent #ENDSARS movement, which had been trending on the social media for a while resulted in nationwide protests on major roads of Nigeria. This protest started on the 2^nd^ of October, 2020 and spanned over three weeks in almost all the states of Nigeria, with some recent resurgence of the protests in some places such as the Federal Capital Territory, Abuja. The protesters are mainly youths who have gathered together to protest the alleged murders, rape, and assault and brutality by a Nigerian Police unit called Special Anti-Robbery Squad (SARS) (Figure 1). The unit was created in 1992 and was supposed to be in charge of robberies, kidnapping, and other violent crimes. Instead, it has been accused of using its position to extort, rob, defraud, injure, and even murder people. Between January 2017 and May 2020, Amnesty International recorded at least 82 cases of “torture, ill treatment and extra-judicial execution” at the hands of SARS agents [7]. The youth have therefore called on the Nigerian government to reform and decentralize the policing system. The protests have over the period garnered solidarity and support from celebrities and individuals within and outside the country with solidarity protests held in Europe, the United Kingdom and United States of America etc. Thus, leading to widespread gathering of youths during the demonstrations. The resultant effect of these have been the breaching of several safety protocols previously recommended by the National Center for Diseases Control (NCDC) [8]. The implications of these breaches with respect to the transmission of infectious diseases such as the COVID-19 pandemic is huge due to the fact that these healthy youths could be undetectable transmitters of the disease in the community.

The WHO guidelines for public health and social measures for slowing or stopping the spread of COVID-19 at national or community level include individual and environmental measures, detecting and isolating cases, contact-tracing and quarantine, social and physical distancing measures including for mass gatherings, international travel measures, vaccines and treatments [6]. The social and physical distancing measures aim to slow the spread of disease, by stopping chains of transmission of COVID-19 and preventing new ones from appearing. These measures secure physical distance between people (of at least one metre), use of facemask and reduce contact with contaminated surfaces, while encouraging and sustaining virtual social connection within families and communities [9]. Measures for the general public include introducing flexible work arrangements such as teleworking, distance learning, reducing and avoiding crowding, closure of non-essential facilities and services, shielding and protection for vulnerable groups, local or national movement restrictions and staying-at home measures, and coordinated reorganization of health care and social services networks to protect hospitals. The measures are used in conjunction with individual protective measures against COVID-19 such as frequent hand washing and cough etiquette and use of face mask in public places [5,9].

**Figure 2:**
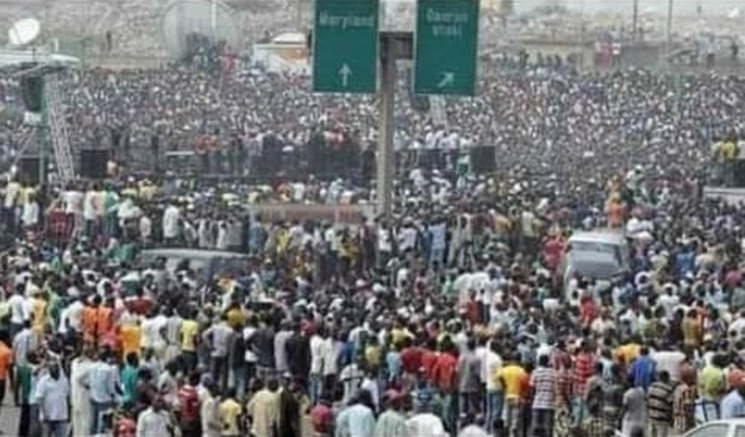
EndSARS Protesters at the Gbagada expressway axis of Lagos state, Nigeria

**Figure 3:**
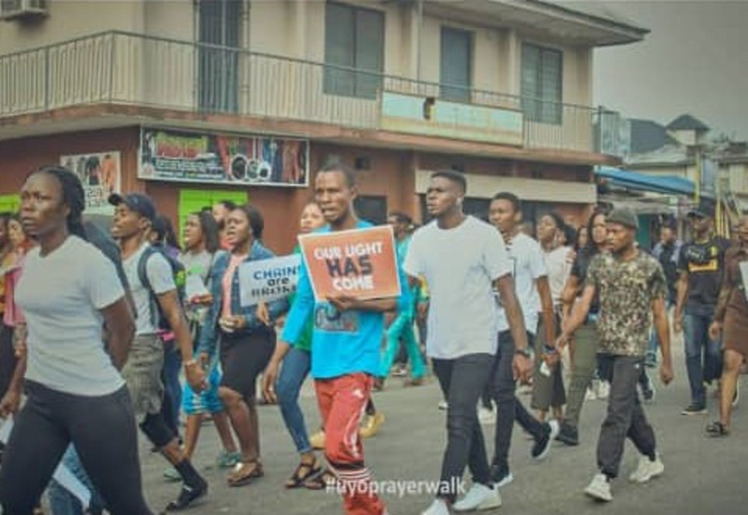
youths engaged in prayer walk during the EndSARS protest in Uyo, Akwa Ibom State, Nigeria

**Figure 4:**
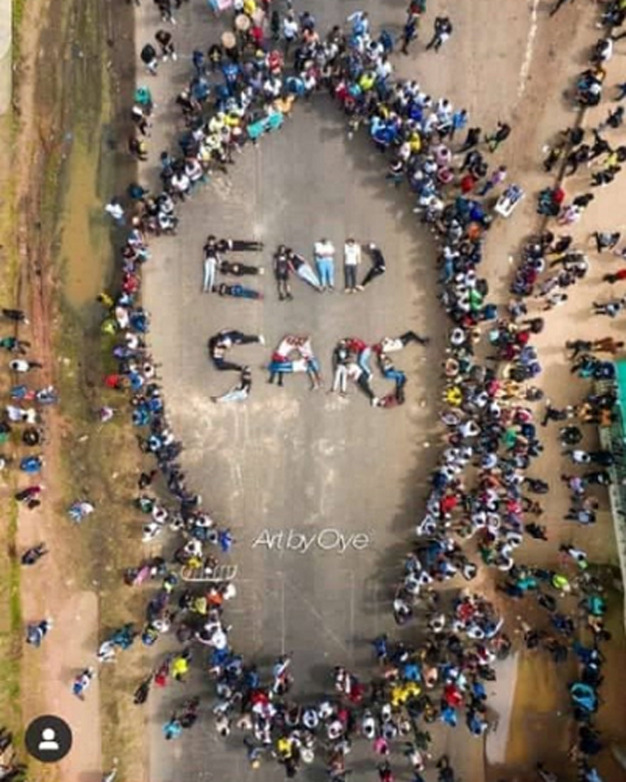
cross-section of youths during the EndSARS protest in Lagos, Nigeria

**Figure 5:**
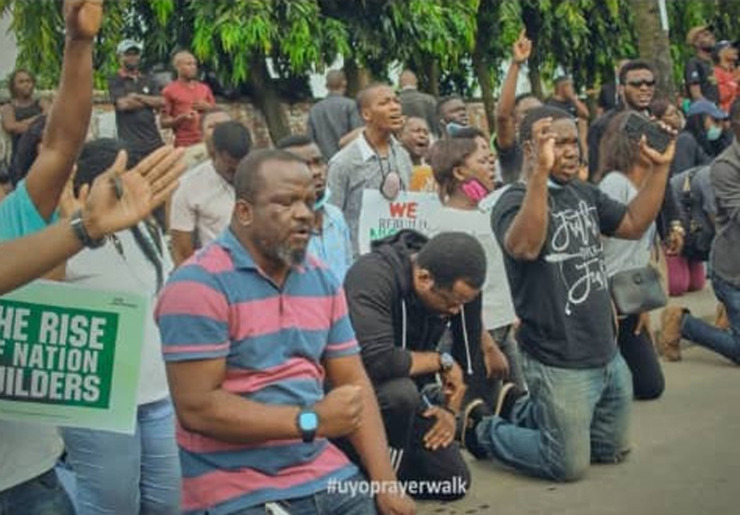
youths engaged in prayer sessions during the EndSARS protest in Nigeria

Unfortunately, with the wake of #EndSARS protest in Nigeria, mammoth crowds of youth have gathered in different places across the country (Figure 2). In these cases, most people flouted most of these COVID-19 public health and social measures which play an essential role in reducing the number of infections and saving lives. The youths were mostly observed without their face/nose masks, crowded together in locations without handwashing points and no hand sanitisers (Figure 2, Figure 3, Figure 4, Figure 5), thereby exposing and increasing the risk of many individuals contracting COVID-19 disease.

In addition to the imposition of curfews and movement restriction put in place to curtail the spread of the protests in several states, consistent awareness was created by the NCDC during the protest [8]. However, it is quite unclear if measures such as screening and testing were escalated or strengthened to help curtail the potential rippling effect of a spike in infections across communities both in Nigeria and other countries especially at the ports of travels.

Recently, there has been reported increase in the number of COVID cases recorded in the country, such that in the weeks just after these protests the country recorded a 32 percent increase (923 cases in the week of 25-31, October, 2020) from the preceding weeks and a subsequent 2 percent increase (in the week of November 1-7, 2020) to 937 new cases [10]. These increases have been recorded in Lagos, FCT, Rivers, Oyo, Ogun, Ekiti, Kwara and Sokoto [5]. Although these figures may seem negligible, given the nature and unpredictable nature of the COVID-19 disease, it is important to take cognizance of these increases to mitigate against possible spike of the diseases curve in the country. This is especially necessary in the face of the fast approaching yuletide period, which is usually characterized with increased travels, mobility and mass gatherings. Futhermore, the full effect of the protest is yet to be felt and there are some potential dire consequences for the continent in the nearest future, due to the fact that Nigeria is the most populous black nation with youths making up over 50% of her population. In addition to this, the impact of travels in and out of the country could pose more risk of community transmission within and outside the country in the aftermath of these protests.

This event has raised the need to engage youth leaders and mobilisers of such demonstrations -as youth influencers- to enlighten the youths on the need to adhere to the prevention guidelines to curb infections that could diminish the gains of nations in the fight against the current pandemic. Given the current realities of the COVID-19 pandemic, there is an urgent need for individuals to be proactive and take responsibility in preventing transmission of the disease. Hence it is important to increase awareness and enforcement of such measures as the use of nose masks, face shields and frequent hand washing or sanitizing in public places. These measures will go a long way to curtail the imminent community transmission of COVID-19 across Nigeria. This situation also calls for more strengthening of these measures put in place by the National Center for Diseases Control (NCDC) especially increasing access to COVID-19 screening services to accommodate the possibility of increased infections in communities within the country.

Furthermore, there is an urgent need for the Africa Centre for Disease Control in collaboration with other stakeholders such as individual National Centers for Disease Control to increase screening facilities and access to these services across regions. Finally, it is strongly recommended that countries collaborate with relevant health agencies such as the World Health Organization (WHO) to develop or strengthen existing contingency plans; revise existing COVID-19 guidelines to factor in potential consequences of such events as conflicts situations, unrest, and protests across countries in Africa and globally.

## References

[1] Liu J, Liao X, Qian S, Yuan J, Wang F, Liu Y (2020). Community Transmission of Severe Acute Respiratory Syndrome Coronavirus 2, Shenzhen, China. Emerg Infect Dis.

[2] Fuk-Woo CJ, Shuofeng Y, Kin-Hang K, Kai-Wang TK, Hin C, Jin Y (2020). A familial cluster of pneumonia associated with the 2019 novel coronavirus indicating person-to-person transmission: a study of a family cluster. Lancet.

[3] European Centre for Disease Prevention and Control COVID-19 situation update worldwide, as of 13 December 2020.

[4] World Health Organisation Coronavirus disease (COVID-19). Data as received by WHO from national authorities, as of 04 October 2020 10 am CEST.

[5] Nigeria Centre for Disease Control and Prevention (NCDC) COVID-19 NIGERIA.

[6] World Health Organisation Coronavirus disease (COVID-19) advice for the public.

[7] TeenVogue EndSARS Movement Is Being Defined by Nigerian Youth.

[8] All Africa Nigeria: Impact of #EndSARS Protests On Nigeria's Covid-19 Status 'Uncertain'.

[9] World Health Organisation Coronavirus disease (COVID-19) advice for the public: When and how to use masks.

[10] All Africa Nigeria Faces Possible Covid-19 Second Wave but Has Let Guard Down.

